# Ultraviolet Laser-induced ignition of RDX single crystal

**DOI:** 10.1038/srep20251

**Published:** 2016-02-05

**Authors:** Zhonghua Yan, Chuanchao Zhang, Wei Liu, Jinshan Li, Ming Huang, Xuming Wang, Guorui Zhou, Bisheng Tan, Zongwei Yang, Zhijie Li, Li Li, Hongwei Yan, Xiaodong Yuan, Xiaotao Zu

**Affiliations:** 1 School of Physical Electronics, University of Electronic Science and Technology of China, Chengdu, 610054, China; 2 Department of Materials Science and Engineering, University of Michigan, Ann Arbor, MI 48109, USA; 3 Research Center of Laser Fusion, China Academy of Engineering Physics, Mianyang 621900, China; 4 Institute of Chemical Materials, China Academy of Engineering Physics, Mianyang 621900, China

## Abstract

The RDX single crystals are ignited by ultraviolet laser (355 nm, 6.4 ns) pulses. The laser-induced damage morphology consisted of two distinct regions: a core region of layered fracture and a peripheral region of stripped material surrounding the core. As laser fluence increases, the area of the whole crack region increases all the way, while both the area and depth of the core region increase firstly, and then stay stable over the laser fluence of 12 J/cm^2^. The experimental details indicate the dynamics during laser ignition process. Plasma fireball of high temperature and pressure occurs firstly, followed by the micro-explosions on the (210) surface, and finally shock waves propagate through the materials to further strip materials outside and yield in-depth cracks in larger surrounding region. The plasma fireball evolves from isotropic to anisotropic under higher laser fluence resulting in the damage expansion only in lateral direction while maintaining the fixed depth. The primary insights into the interaction dynamics between laser and energetic materials can help developing the superior laser ignition technique.

Since being introduced by Brish *et al.*^1^ Menlichelli and Yang^2^ in the late sixties and early seventies of last century, laser ignition technology has received much attentions due to its high precision and safe operation, which has extensive potential applications in the aerospace industry, defense technology, and special processes.

Up to present, most efforts of researchers have been focused on effects of laser parameters, such as wavelength^3^,^4^,^5^, pulse duration (free running or Q-switched mode)^6^,^7^,^8^, radiation-zone diameter^9^, on the initiation energy threshold and fluence of energetic materials. Lisitsyn *et al.*^3^ investigated the behavior of heavy metal azides, AgN_3_ and Pb(N_3_)_2_, irradiated at wavelengths of 1064, 532, 354.7, and 266 nm, respectively, and discussed the reasons through aspects of the bandgap energy width of explosives. Volkova *et al.*^6^ and Aleksandrov *et al.*^7^ explored the influence of pulse width on the initiation energy threshold of PETN and reported that initiation energy density increased with increasing the length of laser pulse. Besides the pulse laser, Ali *et al.* utilized CO_2_ laser ignition experiments^10^ to figure out the reactive mechanisms of HMX and TATB during deflagration-to-detonation transitions. Moreover, additives, such as MgO particles^11^, carbon black^12^,^13^, Al ultradispersed particles, Ni-C and Al-C mechanocomposites^14^, etc. were also used to modulate the laser ignition properties of explosives as the light-scattering media or inclusions for the absorption of laser radiation. Environmental pressure^4^,^15^, surface conditions (open or closed), and the thickness of transparent dielectric^3^,^6^ were found to play important roles in the laser ignition processes.

However, most of the energetic materials used in their works were explosive powders or micro-crystals pressed into tablets or pellets at some pressure. In these traditional sample preparation methods, the chaotic configuration of explosive crystals makes it impossible to establish quantitative investigations on the laser ignition dynamics in aspect of crystallography discrimination. Bulk explosive crystal should be an ideal candidate to investigate the interaction mechanisms between laser and energetic materials. Cyclotrimethylene trinitramine (RDX, hexogen or cyclonite) is one of the high energetic materials widely used in both military and industry^16^. Such colorless crystals that are normally in orthorhombic (P*bca* space groups with a = 1.3182 nm, b = 1.1574 nm, c = 1.0709 nm), can be obtained by various recrystallization techniques such as cooling crystallization, drowning-out, and evaporation crystallization^17^. Generally, there are three typical habits of RDX crystals: equant, prismatic and tabular habits. The prismatic habit enclosed with (210) facets dominated among the larger crystals^16^. Up to present, A. L. Ramaswamy, *et al.*^18^ and Ming-Wei Chen, *et al.*^19^ have explored the fundamental mechanisms of hot spot generation with Nd/glass laser (pulse width of 300 μs) and CO_2_ laser, respectively.

In this work, we present the ultraviolet laser ignition of the predominating (210) facet of prismatic RDX single crystal. The ignition threshold as well as the damage crack growth affected by laser fluence was demonstrated based on the characterizations of optical microscope (OM, NIKON ECLIPSE LV100), scanning electron microscope (SEM, HITACHI S3400 + EDX). The dynamic mechanisms of laser igniting energetic materials were discussed.

## Methods

### Materials

**Figure 1:**
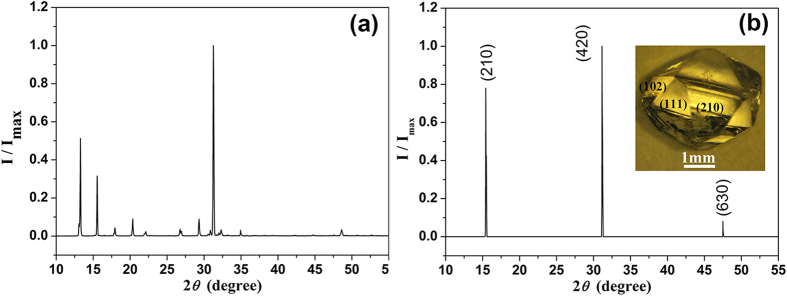
X-ray diffraction of RDX materials: (a) powder crystal (b) (210) facet of bulk crystal and the typical prismatic RDX crystal (inset).

RDX single crystals in this study were prepared by solvent evaporation, spontaneous nucleation, from their saturated solutions in acetone at a constant room temperature. In a typical procedure, 20.0 g RDX was dissolved in 350 mL acetone solvent and the solution was sealed in a 500 mL breaker by a preservative film with several pinholes in order to obtain a slow crystal growth rate. It was then placed in an un-vibrated incubator (SPX-150) for about 35 days at 30 °C with the temperature control precision ± 0.1° C. Figure 1 shows the x-ray diffraction (XRD, DX-2700 and BEDE D1, Cu *K*_*α*_) patterns of RDX powder and bulk crystals, respectively, illustrating the phase purity as well as the crystallography configuration of RDX single crystal as prepared in our work. The inset in Fig. 1b displays the typical prismatic crystal^16^ exhibiting the principal formation of (210), (111), and (102), etc. with the (210) facets dominant. Herein, the (210) facet was chosen as the interaction surface with laser (perpendicular incidence), which had been distinguished with x-ray triple-crystal diffraction method in Fig. 1b.

### Laser ignition systems

**Figure 2:**
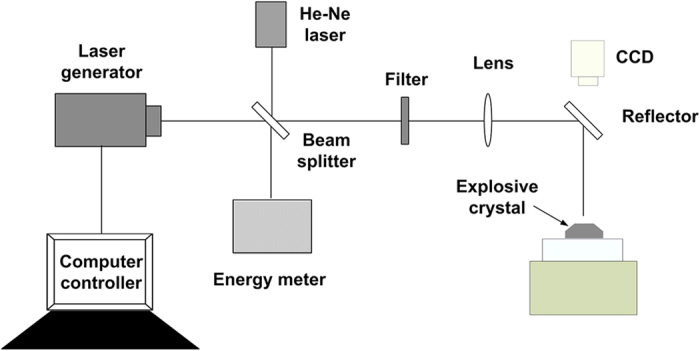
Schematic diagram of experimental devices.

The laser ignition system is schematically demonstrated in Fig. 2. The explosive samples were irradiated by Nd: YAG pulse laser operated at 355 nm with pulse width of 6.4 ns. The beam had a spatially Guassian profile and was focused to 0.4 mm in diameter at 1/e^2^ at the (210) crystal face of RDX crystals. Beam splitter reflected the minority (about 4%) of the beam into the energy meter to monitor laser power. A helium-neon (He-Ne) laser, reflected with the beam splitter and colinear with the incident laser beam, was used to aid in the alignment of the optics and samples. The majority (96%) of laser energy was focused by a lens (50 mm diameter and 1000 mm focal length), coupled with a reflector (HR@355 nm, R > 99%, AOI = 45°), onto the crystal surface to ignite the RDX crystal, which was observed by a CCD online system. Filter was used for the energy reduction when necessary. In our experiments, we focus on the initial process of laser ignition. The experiment platform is designed without constraints for the system simplification. The open atmosphere makes the materials eject during the ignition process, leading to the reaction dying out in the formation of cavities and/or pits (damage regions)^20^. The ignition criterion^18^ is different from that of standard initiation that results in a runaway reaction consuming all of the material.

## Results and Discussion

### Laser ignition threshold studies

**Figure 3:**
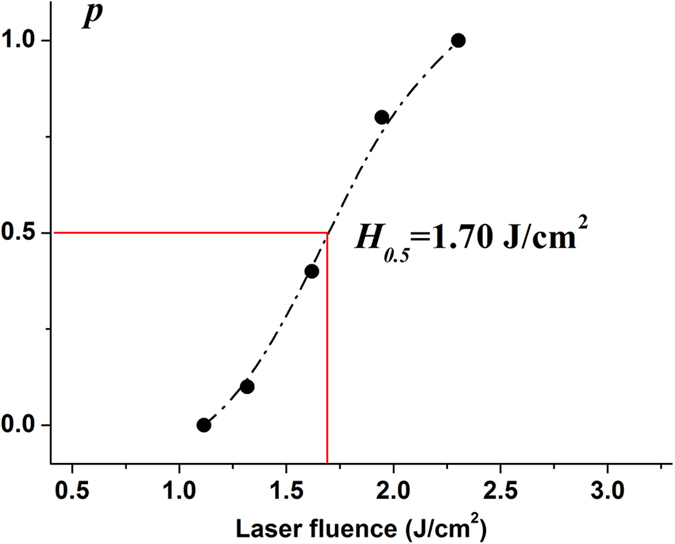
The ignition threshold of RDX crystals.

In consideration of the stochastic nature of the ignition process, the dependence of the probability of explosion incidents, *p*, on the irradiation fluence was utilized to estimate the laser ignition property. As shown in Fig. 3, laser ignition probability of (210) crystal facet of RDX single crystals at 355 nm varies as a function of laser fluences. Every data dot was obtained by recording the number of ignited incidents of RDX crystals among ten events with a single shot at the same laser fluence in different regions of (210) surface. Obviously, the energy threshold of *p* = 0 and 100% can be achieved at fluence of 1.12 J/cm^2^ and 2.30 J/cm^2^, respectively, where RDX single crystals can be hardly and totally initiated. It indicates that 50% probability explosion *H*_*0.5*_ = 1.70 J/cm^2^, which could be used as the laser-induced breakdown threshold in this particular condition^11^.

### Laser-induced damage morphology

**Figure 4:**
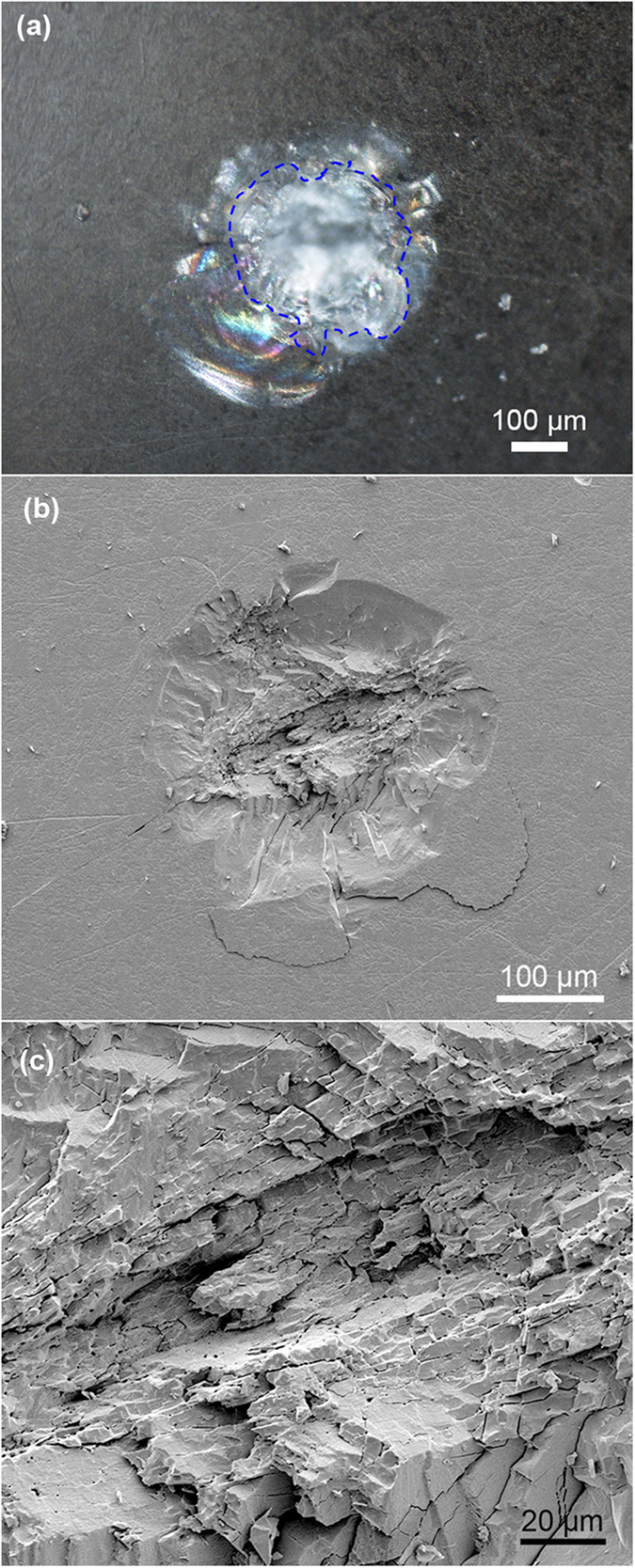
The damage morphologies after laser irradiation at energy fluence of 3.41 J/cm^2^: (a) optical microscope in dark field; (b) overview SEM image of the damage; (c) Magnified of SEM image of the core.

During the laser ignition experiments, plasma fireball and micro-explosion phenomena were clearly observed. Generally, the UV laser initiation of energetic materials can be well explained with photochemical mechanism as reported in references^20^,^21^, in which the stochastic nature of the interaction of photons with the energetic molecules leads to the formation of free radicals that trigger a chain decomposition reaction^20^. At low exposures of laser irradiation, the interaction of photons with the energetic molecules leads to fluctuations of the density of the excited molecules^22^,^23^. These fluctuations are starting to act as nucleation of micro-cells of the explosive decomposition reaction. An increase of the irradiation intensity leads to an increase in the concentration of reaction cells. As irradiation energy is near the initiation threshold, there are plasma fireballs and local micro-explosions by the ejection of materials from the reaction regions. At high exposures of initiation, an explosion of significant part of the sample can be observed. As it is an open atmosphere in our work, and there is no confinement to minimize the energy losses. So the reaction dies out in the formation of damage regions as depicted in Fig. 4. In the following discussions, we investigated the laser-induced damage performance of the (210) facets after laser ignition.

Figure 4a shows the typical optical microscope image in dark field after a single shot at the laser fluence of 3.41 J/cm^2^ over the laser-induced breakdown threshold. The explosive material is fiercely damaged after laser irradiation and the optical interference from the produced cracks makes the appearance ambiguous in central shot region, while demonstrating the in-depth expansion of cracks under the surface. The scanning electron microscope (SEM) result in Fig. 4b gives more direct presentation of the surface morphology, corresponding to the region marked with blue short dashes in Fig. 4a. The damage area appears to be dominated by fracture instead of melting, which should be associated with material spallation. The damage morphology of RDX crystal is consisted of two distinct regions: a core region of layered fracture resulted from absorbing the most energies of laser irradiation and a peripheral region of stripped material surrounding the core, indicating mechanical damage accompanied by micro-explosions. Simultaneously, some fragments eject outside from the core, which can be also verified in Fig. 4a. By comparing Fig. 4a,b, it proves the existence of in-depth cracks extending inside the RDX crystals except for aforesaid surface damages, which are not directly displayed in the SEM owing to its focus-on surface characteristic. These in-depth cracks should originate from the shock wave propagation right after the laser ignition, which change the reflectivity of detection light in the optical microscope. This fact accounts for the bright interference contrast of outside region in Fig. 4a.

Figure 4c shows a clear SEM image of the core damage region. It reveals that the core is loose and filled with oriented fractures and blocks, significantly different from the surrounding smooth cleavage surfaces. It could be ascribed to synergetic interaction between the strong breakdowns of plasma/micro-explosion and the distinct crystal orientation, which makes the materials can not cleave successively along the inherent cleavage planes of RDX crystal in the core region like what the surrounding region does.

### Damage area and depth

**Figure 5:**
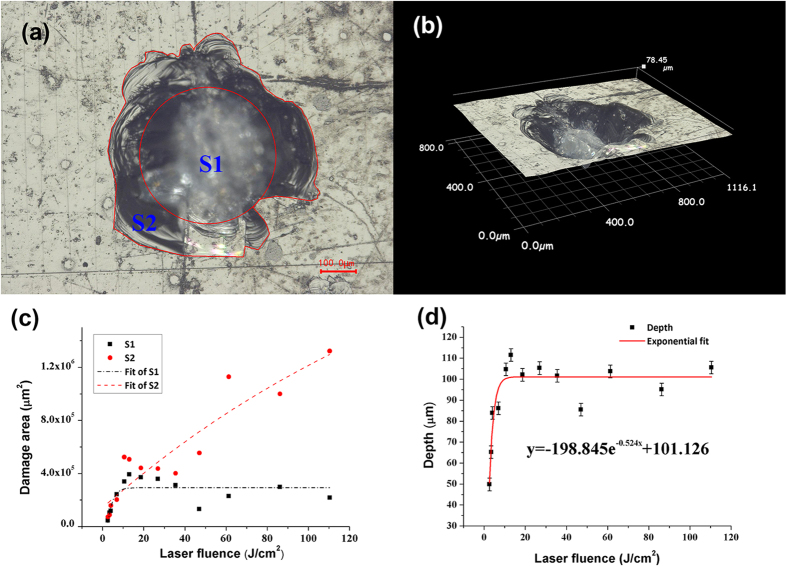
Damage morphologies and dimensions: (a) 2D and (b) 3D optical images of damage morphology after a single shot at 4.02 J/cm^2^; (c) damage area and (d) depth as a function of laser fluence.

To put further insights into the dynamic details of laser-induced ignition process, the growth manner of damage region driven by the increase of laser fluence has been quantitatively studied. The damage areas and depths at laser fluence levels from 2.6 to 120 J/cm^2^ were all measured. Figure 5a depicts a typical two-dimensional (2D) damage morphology after a single shot at the laser fluence of 4.02 J/cm^2^. For convenience, symbols “S1” and “S2” are applied to represent the core region of layered fracture and the surrounding region of stripped materials (whole damage region except S1), respectively. For the depth measurements, three-dimensional pattern of the damage in Fig. 5a is reconstructed by a digital microscope (KEYENCE VHX-2000), in which the depth is defined as the distance from the crystal surface to the bottom of the core.

Figure 5c,d illustrate the profiles of damage area and depth changing with increasing the laser fluence. It demonstrates that both the area of “S1” and “S2” play approximately equal roles in the damage region at the lower level of laser fluence, while above the level of 40 J/cm^2^, the crack area “S2” does the most. As the laser fluence increases, damage area of “S1” increases firstly, and then remain in an approximately constant value when the laser fluence is over 12 J/cm^2^. While the crack area “S2” increases with the increasing fluence all the time.

The distinct damage growth behaviors indicate significant dynamic details of laser-induced ignition procedure. Specially, when the laser intensity is low but above the ignition threshold, not the whole but the central region of irradiated material can be ignited due to the gradient energy dispersion of incident Gaussian laser beam. As the laser intensity increases, the ignited regions in irradiated material increase. When all the regions in irradiated material can be ignited (above 12 J/cm^2^), the damage area of the core will not increase with the increasing fluence any more due to the limitation of beam diameter. However, the plasma fireballs, micro-explosions of energetic crystals, and the subsequent shock waves are always enhanced with increasing fluence during the laser ignition process, which leads to more serious damages to the surrounding materials and makes this region expand forward. Besides, the damage depth has the similar tendency like “S1”. It is quite intriguing to discover that the damage depth does not grow with laser fluence increasing but saturate at certain value. This suggests that as the laser fluence increases, plasma-ball grows rapidly in size. At the beginning with increasing fluence, it increases approximatively as an isotropic ball, while it evolves to be anisotropic when the fluence is larger, and finally ceases its expansion in longitudinal direction. When the laser energy increases, RDX crystals behind the plasma front are shielded from the remnant energy of laser pulse, which keeps the damage depth in the range about 100 microns at the fluence above 12 J/cm^2^, as the equation described in Fig. 5d.

From the distinct evolution behaviors of damage morphology demonstrated above, it can be deduced that during the laser ignition, the plasma fireball of high temperature and pressure occurs firstly, which is followed by the physical-chemical micro-explosions on the (210) surface. Finally, shock waves, resulting from the micro-explosion, propagate through the materials to further strip materials outside and yield in-depth cracks in larger surrounding region.

## Conclusions

In this work, the (210) crystal facets of secondary explosive RDX single crystals were ignited by ultraviolet laser (355 nm, 6.4 ns) pulses with fluences above the laser-induced breakdown threshold of 1.7 J/cm^2^, and their damage growth manners were characterized in detail. Experimental evidences reveal that the laser-induced damage of RDX crystal can be dynamically defined into two distinct regions: a core region of layered fractures resulted from absorbing the most energies of incident laser and an outside region of stripped material surrounding the core. As laser fluence increases, the area of the crack region, “S2”, increases all the way, while both the area of the core region, “S1”, and the depth of damage increase firstly, and then remain in an approximately constant value when the laser fluence is over 12 J/cm^2^. These quantitative results suggest that laser-induced plasma fireball occurs firstly, followed by the micro-explosions on (210) surface of RDX, and the as produced shock waves resulting from the micro-explosion propagate outwards through the materials. The plasma fireball behaves in an anisotropic manner, it increases isotropically at the beginning with increasing fluence, while evolves into an elliptical shape without further expansion in longitudinal direction when the fluence is larger. The above dynamics would account for the distinct damage morphology evolution as the laser fluence increases. This work offers a primary inspiration for better understanding the interaction dynamics between laser and energetic materials.

## Additional Information

**How to cite this article**: Yan, Z. *et al.* Ultraviolet Laser-induced ignition of RDX single crystal. *Sci. Rep.* **6**, 20251; doi: 10.1038/srep20251 (2016).
